# Comparison of laryngeal mask airway and endotracheal tube in preterm neonates receiving general anesthesia for inguinal hernia surgery: a retrospective study

**DOI:** 10.1186/s12871-021-01418-2

**Published:** 2021-07-21

**Authors:** Miao-Pei Su, Ping-Yang Hu, Jao-Yu Lin, Shu-Ting Yang, Kuang-I Cheng, Chia-Heng Lin

**Affiliations:** 1 Department of Anesthesiology, Kaohsiung Medical University Hospital, Kaohsiung Medical University, No.100, Tzyou 1st Rd, Kaohsiung, Taiwan; 2 Division of Pediatric Surgery, Department of Surgery, Kaohsiung Medical University Hospital, Kaohsiung Medical University, Kaohsiung, Taiwan; 3 Division of Neonatology, Department of Pediatrics, Kaohsiung Medical University Hospital, Kaohsiung Medical University, Kaohsiung, Taiwan; 4 School of Medicine, College of Medicine, Kaohsiung Medical University, Kaohsiung, Taiwan

**Keywords:** Laryngeal mask airway, Premature, Hernia repair, General anesthesia

## Abstract

**Background:**

Preterm neonates are at higher risk of developing inguinal hernia, and have an increased risk of perioperative adverse events. Laryngeal mask airway (LMA) is claimed to be associated to decreasing perioperative respiratory complications compared to endotracheal tube (ETT) in infants under one year of age receiving minor surgery; thus, we conducted a retrospective survey in former preterm neonates below 5000 g to compare the respiratory complications between LMA and ETT in general anesthesia for inguinal hernia surgeries.

**Methods:**

The inclusion criteria were: gestational age at birth under 37 weeks, body weight at surgery below 5000 g, and receiving scheduled inguinal hernia repair under general anesthesia with LMA or ETT. Infants who were dependent on mechanical ventilation preoperatively were excluded. The postoperative respiratory complications including delayed extubation, re-intubation, and apnea within postoperative 24 h were compared between groups.

**Results:**

From July 2014 to December 2017, 72 neonates were enrolled into final analysis. There were 57 neonates managed with LMA, and only 15 neonates intubated with ETT during the study period. The gestational age at birth and post-menstrual age at surgery showed no significant difference between groups, although in the ETT group, the body weight at birth and at surgery were lower, and more infants had history of severe respiratory distress syndrome and had received oxygen therapy within two weeks prior to surgery. Surprisingly, none one of the infants developed delayed extubation, re-intubation, or postoperative apnea in the LMA group. In the ETT group, 40 percent of the neonates could not be successfully extubated in the operation theater.

**Conclusion:**

In preterm neonates, even in those younger than 52 weeks post-menstrual age who undergoing inguinal hernia repair in their early infancy, LMA appears feasible and safe as the airway device during general anesthesia in specific patient group. However, anesthesiologist might prefer ETT rather than LMA in some complex situation. In neonates with lower body weight at birth and at surgery, and with a history of severe RDS and oxygen-dependence, further prospective study is required.

## Background

Inguinal hernia is a common surgical disease in former preterm infants. The incidence of inguinal hernia is 9 to 11% and is especially higher in very preterm infants and low birth weight infants [1–3]. Low birth weight, lung disease, and history of ventilator support are all risk factors for inguinal hernia, and these factors are also associated with perioperative respiratory adverse events.

In previous studies, general anesthesia was considered as related to higher perioperative adverse events such as apnea and bradycardia compared to spinal anesthesia in preterm infants receiving inguinal hernia repairs [4–8]. The endotracheal tube has traditionally been used for general anesthesia in infants to secure the airway and provide ventilation support if needed; however, since the laryngeal mask airway (LMA) is being increasingly used in pediatric anesthesia, neonatal resuscitation and surfactant administration in preterm neonates, it is time to evaluate the role of LMA as an airway device for general anesthesia in preterm neonates.

In a large randomized control trial in infants (aged under 12 months) receiving minor surgery, LMA was related to decreasing the occurrence of perioperative respiratory adverse events compared to those with endotracheal tubes (ETT) [9]. However, the mean age at surgery was above six months. In surgery for retinopathy of premature, the suggestion of using LMA in general anesthesia for preterm neonates is controversial [10, 11]. Thus, we conducted a retrospective survey, focused on preterm neonates undergoing hernia surgery, to compare the influence of LMAs and ETTs on postoperative respiratory complications. The hypothesis was that preterm neonates below 5000 g receiving LMAs had lower incidence of postoperative respiratory complications compared to those with ETTs.

## Methods

This retrospective study involved chart review from July 2014 to December 2017 of neonates undergoing inguinal hernia repair in Kaohsiung Medical University Hospital, and was approved by the Kaohsiung Medical University Hospital Institutional Review Board (KMUHIRB-SV(I)-20180005).

The inclusion criteria were as follows: body weight at operation below 5000 gm, gestational age at birth under 37 weeks, and elective inguinal hernia repair under general anaesthesia with LMA or ETT. Infants who were intubated or dependent on mechanical ventilation preoperatively; lacked birth or hospital records; had received other major surgery involving vital organ concomitantly, and had major cardiopulmonary diseases were all excluded. Those receiving minor surgeries usually accompanied with inguinal hernia repairs (eg. orchiopexy or hydrocelectomy) were not excluded.

Pediatric inguinal herniorraphy is routinely performed with an open inguinal approach under general anesthesia in our institution, without regional block, with the anesthesia being induced and maintained with sevoflurane. Neuromuscular blocking agents are routinely not applied for LMA insertion in our institution; as for endotracheal intubation, it depends on each anesthesiologist’s decision. The choice of airway device for general anesthesia was dependent on the preference of individualized anesthesiologist. In this study, patients were allocated into either the LMA or ETT group according to the airway device used during the maintenance of anesthesia. These surgeries were performed by two pediatric surgeons and both of them did not perform contralateral exploration for clinically occult hernia in contralateral side.

Demographic data including gestational age at birth, post-menstrual age at surgery, body weight at birth and at surgery, Apgar score at birth, history of severe respiratory distress syndrome (RDS) and neonatal apnea, pharmacologic history of surfactant administration and methylxanthines, preoperative oxygen-free days, patient origin, and the time duration of operation and anesthesia were documented. Grades 3 and 4 RDS were recorded as severe RDS. If RDS was documented in the medical records without grading, an independent neonatologist blinded to the study group reviewed and graded the first chest radiography after birth as to the severity of RDS. In our institution, the available methylxanthines for RDS treatment are only theophylline and aminophylline, with caffeine being unavailable. The preoperative oxygen-free days indicated the days the neonate was totally independent from any oxygen supplementation. The patient origin indicates the surgery was arranged from the neonatal intensive care unit (NICU) / complete neonatal care unit (CNU) or from pediatric surgery outpatient department (OPD).

The outcomes were delayed extubation, re-intubation, and postoperative apnea, and the length of post-operative stay Delayed extubation was defined as the airway device remaining in situ while the patient left the operation room; re-intubation was defined as the infant being extubated postoperatively and then re-intubated within postoperative 24 h; while postoperative apnea was defined as apnea recorded in NICU within postoperative 24 h.

Pearson Chi-square test or Fisher’s exact test was performed for categorical data; Student's t-test was used for continuous data; and Mann–Whitney U test was applied for data not normally distributed, with p values less than 0.05 considered statistically significant.

## Results

**Fig. 1:**
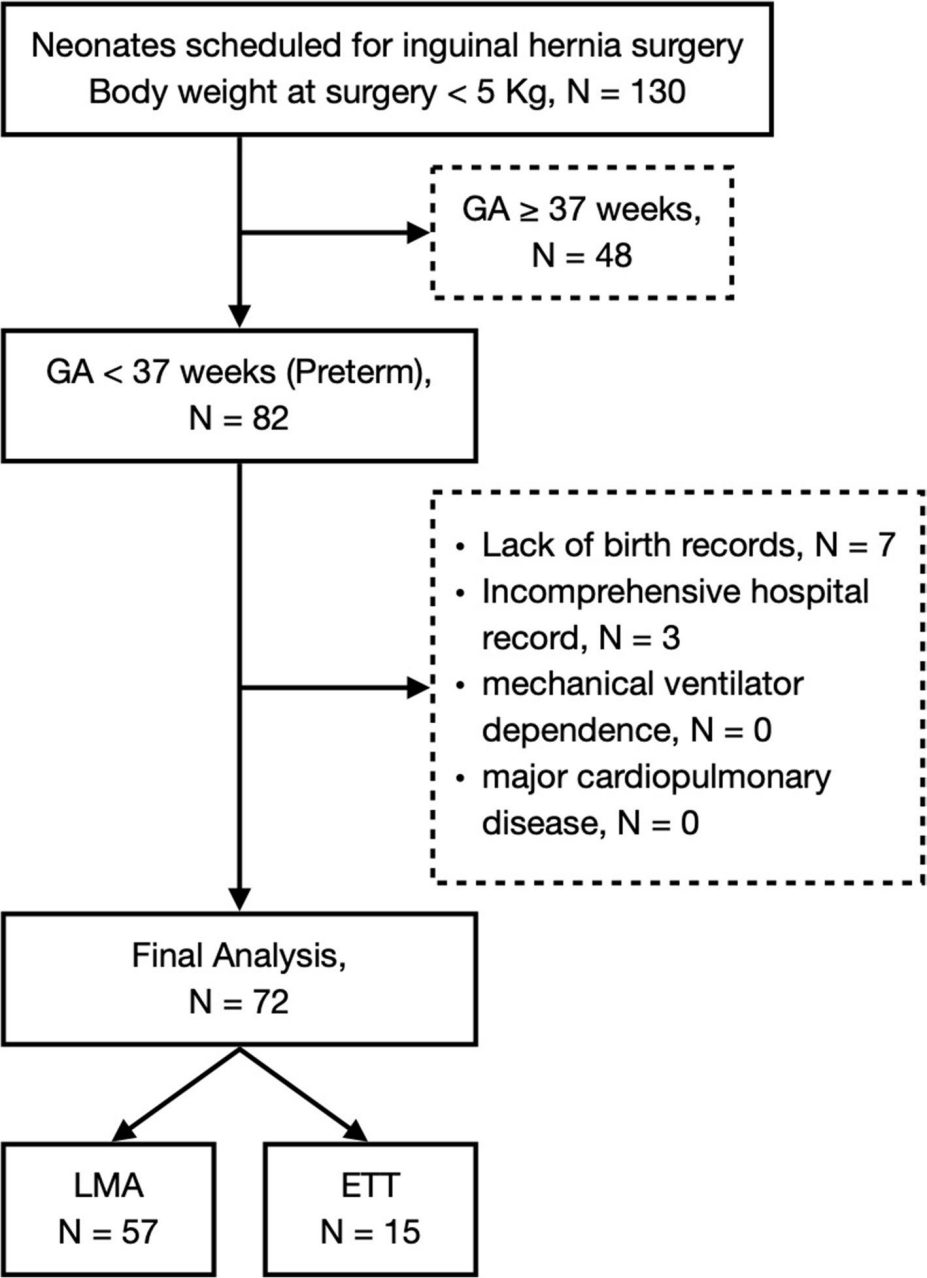
Flow diagram. GA: general anesthesia

Among the 130 neonates weighing below 5000 gm receiving inguinal hernia repairs during the chart reviewal period, 48 of them were term or full-term infants. Of the remaining 82 preterm neonates, seven lacked full birth records due to transfers from other hospitals and three had incomprehensible hospital records. None of them was dependent on mechanical ventilator just prior to surgery or received other major surgery concomitantly. Thus, 72 neonates were enrolled into final analysis (Fig. 1).

According to the airway device used during the maintenance of anesthesia, there were 57 in the LMA group and 15 in the ETT group. In the ETT group, one patient was intubated due to poor ventilation with LMA during induction of anesthesia, and 11 out of 15 patients received intravenous cisatracurium (ranged 0.05 – 0.25 mg) during induction of anesthesia to facilitate endotracheal intubation. Males predominated only in the LMA group (Table 1).

**Table 1:** Patient Demography

	LMA, *n* = 57	ETT, *n* = 15	*p* value
Male	41 (71.9%)	5 (33.3%)	0.006
Gestational age, birth (week)	32 (29 – 35)	31 (27 – 34)	0.223
PMA, surgery (week)	39 (36 – 42)	38 (37 – 39)	0.137
Weight, birth (gm)	1745 (1259 – 2230)	1188 (803 – 1710)	0.009
Weight, surgery (gm)	2630 (2265 – 3775)	2300 (2130 – 2600)	0.009
Severe RDS	11 (19.3%)	7 (46.7%)	0.044
Medication history			
Surfactant	6 (10.5%)	1 (6.7%)	1.000
Aminophyline	17 (30.0%)	6 (40.0%)	0.537
Theophyline	14 (24.6%)	7 (46.7%)	0.116
Oxygen free > 2 weeks	52 (91.2%)	8 (53.3%)	0.002
Birth History			
Cesarean delivery	32 (56.1%)	8 (53.3%)	0.846
Apgar 1”	6.12 ± 2.07	6.00 ± 1.92	0.836
Apgar 5”	7.74 ± 1.72	7.53 ± 0.13	0.699
Patient origin			
OPD	31 (54.4%)	3 (20%)	0.018
NICU/CNU	26 (45.6%)	12 (80%)	

Data are presented as n (%), median (IQR), or mean ± SD

*PMA* Post-menstrual age, *RDS* Respiratory distress syndrome, *OPD* Out-patient department, *NICU* Neonatal intensive care unit, *CNU* Complete neonatal care unit

The median gestational age at birth (LMA 32 weeks vs. ETT 31 weeks) and the post-menstrual age at surgery (LMA 39 weeks vs. ETT 38 weeks) showed no significant differences between groups. The maximum post-menstrual age was 52 weeks in the LMA group and 41 weeks in ETT group; however, the median body weights at birth and at surgery in the ETT group were significantly lower than in the LMA group (1188 gm vs. 1745 gm at birth, and 2300 gm vs. 2630 gm at surgery, respectively).

The proportion of severe RDS in the ETT group was also higher than in the LMA group. About half of the neonates (38 out of 72, 52.8%) included in this study were diagnosed with severe RDS, and one of them developed moderate bronchopulmonary dysplasia. Most of the patients in the LMA group were free from oxygen supplementation for more than two weeks (LMA 91.2% vs. ETT 53.3%, p = 0.002). The medication history related to RDS treatment, including the usage of surfactant, aminophylline, and theophylline, revealed no difference between the two groups (Table 1).

In the birth history, there was no difference in the way of delivery (Cesarean delivery or vaginal birth), and in Apgar score at 1 and 5 min between two groups (Table 1). As for the patient origin, 34 surgeries were arranged in the pediatric surgical outpatient department, and the other 38 were transferred from the NICU or CNU. In those from the pediatric surgical outpatient department, above 90 percent of the preterm infants were managed with LMA during general anesthesia.

More patients in the ETT group received bilateral surgery (LMA 31.6% vs. ETT 73.3%, p < 0.01) (Table 2). The median surgical time (LMA 20 min vs. ETT 25 min) and the median anesthesia time (LMA 35 min vs. ETT 50 min) were both significantly longer in the ETT group.

**Table 2:** Intraoperative Parameters

	LMA, *n* = 57	ETT, *n* = 15	*p* value
Bilateral surgery	18 (31.6%)	11 (73.3%)	0.003
Surgical time (min)	20 (20 – 25)	25 (20 – 35)	0.004
Anesthesia time (min)	35 (30 – 45)	50 (40 – 70)	0.034

Data are presented as n (%) or median (IQR)

In the LMA group, no patient experienced delayed extubation, re-intubation, or postoperative apnea after surgery. In the ETT group, six neonates had delayed extubation and were dependent on mechanical ventilator support while leaving the operation room (Table 3). All of the six patients received intravenous cisatracurium during induction of anesthesia. Two were extubated within one hour after the surgery, three during postoperative 1 to 12 h, and the other one at postoperative 24 h. Re-intubation was required in only one case in the ETT group, and she was re-intubated immediately for desaturation in the operation room after extubation, and then extubated successfully at postoperative 6 h in the NICU. Postoperative apnea was noted in two cases in the ETT group, with both of them being also delayed extubated. One was extubated at postoperative 6 h, and the apnea was observed only on the first day, with the other being extubated at postoperative 24 h, and the episodes of apnea remained for three days postoperatively.

**Table 3:** Postoperative Respiratory Complications

	LMA, *n* = 57	ETT, *n* = 15	*p* value
Delayed extubation	0 (0%)	6 (40%)	< 0.001
Re-intubation	0 (0%)	1 (6.7%)	0.208
Postoperative apnea	0 (0%)	2 (13.3%)	0.041

Data are presented as n (%)

The postoperative hospital stay was significantly shorter in the LMA group (LMA 2.9 ± 3.94 days vs. ETT 9.2 ± 8.97 days, p = 0.018). All of the infants enrolled in this study were hospitalized after surgery at least overnight, because the high risk of postoperative apnea in infants under 60 weeks post-menstrual age was against our ambulatory setting [12].

## Discussion

This retrospective study represented a significantly lower incidence of delayed extubation in preterm neonates receiving LMAs as the airway device for general anesthesia compared to ETT in hernia repair surgery. Despite the difference in gender, in respiratory history, and in body weight at birth and at the surgical time between the two groups, LMA serves as a good choice for general anesthesia in preterm neonates undergoing inguinal hernia surgery. The incidence of delayed extubation in preterm or high-risk infants is 17.2% to 35.7% in different reports [6, 7, 13]; this variation could depend on the definition of delay, patient characteristics, anesthetic agents used, and whether muscle relaxant was used or not. Many studies have suggested that regional anesthesia is preferred rather than general anesthesia in preterm infants receiving hernia surgery in early infancy for lower postoperative respiratory complications, but these comparisons mainly focused on the general anesthesia with endotracheal intubation [8, 14, 15]. In a large randomized control trial, LMAs were related to lower perioperative respiratory adverse events in infants below one year of age receiving minor elective surgery [9]. In our report, the use of LMAs in anesthesia for premature infants under 52 weeks of post-menstrual age could also be taken into consideration for the possible lower incidence of postoperative prolonged mechanical ventilator dependence.

There is increasing evidence for the feasibility of LMA placement in newborns. In neonatal resuscitation, LMA is reported as being more effective than the face mask in providing ventilation and preventing further endotracheal intubation in several small randomized controlled trials [16–18]. In surfactant therapy for preterm newborns weighing above 1000 gm with moderate RDS, administration through LMA appears related to lower mechanical ventilation requirement compared to administration through ETT [19, 20]. In infants aged between 29 to 35 weeks of gestational age, Wanous et al*.* reported that there were only slightly changes in heart rate and oxygen saturation during LMA placement for surfactant administration [21]. In the LMA group in our retrospective data, the median post-menstrual age at surgery was 39 weeks (range 37 – 42 weeks), and the median weight at surgery was 2630 gm (range 2280 – 3750 gm). LMA placement in this subgroup of preterm neonates appears feasible.

To our knowledge, this is the first report describing the application of LMA in preterm infants receiving general anesthesia for hernia surgeries in their early infancy. In our institution, anesthesiologists choose LMA as the airway device in most children and infants receiving hernia repair based on the belief that LMA is associated with fewer perioperative respiratory adverse events and is less time-consuming for intubation, especially in patients arranged from OPD, even though their post-menstrual age is under 60 weeks. But in those referred from the NICU and CNU however, 80% of the preterm neonates were anesthetized with ETT intubation. The clinical factors influencing the anesthesiologist’s choice between the two airway devices might include body weight at surgery and at birth, history of severe respiratory distress syndrome, oxygen dependence history, patient origin, and bilateral surgery. Whether these factors affect the safety of LMA used in neonatal anesthesia requires further research.

There are some limitations in this study. Firstly, since difference in patient characteristics between the two groups existed in this retrospective survey, we could only conclude that LMA might be related to less postoperative respiratory complications then ETT in a specific subgroup of preterm neonates receiving general anesthesia, and further randomized study is needed. Secondly, there was a total of 15 attending anesthesiologists involved in this study population, and individualized decisions from each anesthesiologist might present selection bias to the clinical outcomes. Thirdly, although regional anesthesia was widely applied in inguinal hernia repairs in neonates, these surgeries were routinely performed under general anesthesia in our institution, because both pediatric surgeons in our institution requested for avoidance of any sudden patient movement during surgery. Finally, the small sample size, especially in the ETT group, might limit the validity of the conclusions.

## Conclusion

In preterm neonates undergoing inguinal hernia repair in their early infancy, even among those younger than 52 weeks post-menstrual age, LMA appears feasible and safe as the airway device during general anesthesia in specific patient group. However, anesthesiologist might prefer ETT rather than LMA in some complex situation. In neonates with lower body weight at birth and at surgery, a history of severe RDS, and having recently received oxygen therapy, further prospective study is needed.

## Data Availability

The datasets during and/or analyzed during the current study is available from the corresponding author on reasonable request.

## Abbreviations

LMT: Laryngeal mask airway
ETT: Endotracheal tube
RDS: Respiratory distress syndrome
NICU: Neonatal intensive care unit
CNU: Complete neonatal care unit
OPD: Outpatient department

## References

[1] Peevy KJ, Speed FA, Hoff CJ (1986). Epidemiology of inguinal hernia in preterm neonates. Pediatrics.

[2] Grosfeld JL (1989). Current concepts in inguinal hernia in infants and children. World J Surg.

[3] Fu YW, Pan ML, Hsu YJ, Chin TW (2018). A nationwide survey of incidence rates and risk factors of inguinal hernia in preterm children. Pediatr Surg Int.

[4] Welborn LG, Rice LJ, Hannallah RS, Broadman LM, Ruttimann UE, Fink R (1990). Postoperative apnea in former preterm infants: prospective comparison of spinal and general anesthesia. Anesthesiology.

[5] Krane EJ, Haberkern CM, Jacobson LE (1995). Postoperative apnea, bradycardia, and oxygen desaturation in formerly premature infants: prospective comparison of spinal and general anesthesia. Anesth Analg.

[6] Somri M, Gaitini L, Vaida S, Collins G, Sabo E, Mogilner G (1998). Postoperative outcome in high-risk infants undergoing herniorrhaphy: comparison between spinal and general anaesthesia. Anaesthesia.

[7] Kim GS, Song JG, Gwak MS, Yang M (2003). Postoperative outcome in formerly premature infants undergoing herniorrhaphy: comparison of spinal and general anesthesia. J Korean Med Sci.

[8] Dohms K, Hein M, Rossaint R, Coburn M, Stoppe C, Ehret CB (2019). Inguinal hernia repair in preterm neonates: is there evidence that spinal or general anaesthesia is the better option regarding intraoperative and postoperative complications? A systematic review and meta-analysis. BMJ Open.

[9] Drake-Brockman TFE, Ramgolam A, Zhang G, Hall GL, von Ungern-Sternberg BS (2017). The effect of endotracheal tubes versus laryngeal mask airways on perioperative respiratory adverse events in infants: a randomised controlled trial. The Lancet.

[10] Pramod V, Milind J, Preety S (2013). Use of laryngeal mask airway in premature infant. Indian J Anaesth.

[11] Renu S, Praveen T, Rashmi R, Rajvardhan A, Virender KM (2014). Perioperative management and post-operative course in preterm infants undergoing vitreo-retinal surgery for retinopathy of prematurity: A retrospective study. J Anaesthesiol Clin Pharmacol.

[12] Massoud M, Kuhlmann AYR, van Dijk M, Staals LM, Wijnen RMH, van Rosmalen J (2019). Does the Incidence of Postoperative Complications After Inguinal Hernia Repair Justify Hospital Admission in Prematurely and Term Born Infants?. Anesth Analg.

[13] Gurria J, Kuo P, Kao A, Christensen L, Holterman A (2017). General endotracheal vs. non-endotracheal regional anesthesia for elective inguinal hernia surgery in very preterm neonates: a single institution experience. J Pediatr Surg.

[14] Davidson AJ, Morton NS, Arnup SJ, de Graaff JC, Disma N, Withington DE (2015). Apnea after Awake Regional and General Anesthesia in Infants: The General Anesthesia Compared to Spinal Anesthesia Study-Comparing Apnea and Neurodevelopmental Outcomes, a Randomized Controlled Trial. Anesthesiology.

[15] Jones LJ, Craven PD, Lakkundi A, Foster JP, Badawi N (2015). Regional (spinal, epidural, caudal) versus general anaesthesia in preterm infants undergoing inguinal herniorrhaphy in early infancy. Cochrane Database Syst Rev.

[16] Zhu XY, Lin BC, Zhang QS, Ye HM, Yu RJ (2011). A prospective evaluation of the efficacy of the laryngeal mask airway during neonatal resuscitation. Resuscitation.

[17] Trevisanuto D, Cavallin F, Nguyen LN, Nguyen TV, Tran LD, Tran CD (2015). Supreme laryngeal mask airway versus face mask during neonatal resuscitation: a randomized controlled trial. J Pediatr.

[18] Qureshi MJ, Kumar M (2018). Laryngeal mask airway versus bag-mask ventilation or endotracheal intubation for neonatal resuscitation. Cochrane Database Syst Rev.

[19] Pinheiro JM, Santana-Rivas Q, Pezzano C (2016). Randomized trial of laryngeal mask airway versus endotracheal intubation for surfactant delivery. J Perinatol.

[20] Barbosa RF, Simoes ESAC, Silva YP (2017). A randomized controlled trial of the laryngeal mask airway for surfactant administration in neonates. J Pediatr (Rio J).

[21] Wanous AA, Wey A, Rudser KD, Roberts KD (2017). Feasibility of Laryngeal Mask Airway Device Placement in Neonates. Neonatology.

